# MultiComponent Exercise and theRApeutic lifeStyle (CERgAS) intervention to improve physical performance and maintain independent living among urban poor older people - a cluster randomised controlled trial

**DOI:** 10.1186/s12877-015-0002-7

**Published:** 2015-02-11

**Authors:** Debbie Ann Loh, Noran Naqiah Hairi, Wan Yuen Choo, Farizah Mohd Hairi, Devi Peramalah, Shathanapriya Kandiben, Pek Ling Lee, Norlissa Gani, Mohamed Faris Madzlan, Mohd Alif Idham Abd Hamid, Zohaib Akram, Ai Sean Chu, Awang Bulgiba, Robert G Cumming

**Affiliations:** 1 Julius Centre University of Malaya, Department of Social and Preventive Medicine, Faculty of Medicine, University of Malaya, Kuala Lumpur, Malaysia; 2 Centre for Population Health (CePH), Department of Social and Preventive Medicine, Faculty of Medicine, University of Malaya, Kuala Lumpur, Malaysia; 3 Department of Oral Pathology, Oral Medicine and Periodontology, Faculty of Dentistry, University of Malaya, Kuala Lumpur, Malaysia; 4 Fitness First Malaysia, Menara MBF, Jalan Sultan Ismail, Kuala Lumpur, Malaysia; 5 Centre for Education and Research on Ageing, Concord Hospital, Concord, NSW Australia; 6 Sydney School of Public Health, University of Sydney, Sydney, NSW Australia

**Keywords:** Elderly, Exercise, Physical function, Frailty, Randomised controlled trial, Lifestyle

## Abstract

**Background:**

The ability of older people to function independently is crucial as physical disability and functional limitation have profound impacts on health. Interventions that either delay the onset of frailty or attenuate its severity potentially have cascading benefits for older people, their families and society. This study aims to develop and evaluate the effectiveness of a multiComponent Exercise and theRApeutic lifeStyle (CERgAS) intervention program targeted at improving physical performance and maintaining independent living as compared to general health education among older people in an urban poor setting in Malaysia.

**Methods:**

This cluster randomised controlled trial will be a 6-week community-based intervention programme for older people aged 60 years and above from urban poor settings. A minimum of 164 eligible participants will be recruited from 8 clusters (low-cost public subsidised flats) and randomised to the intervention and control arm. This study will be underpinned by the Health Belief Model with an emphasis towards self-efficacy. The intervention will comprise multicomponent group exercise sessions, nutrition education, oral care education and on-going support and counselling. These will be complemented with a kit containing practical tips on exercise, nutrition and oral care after each session. Data will be collected over four time points; at baseline, immediately post-intervention, 3-months and 6-months follow-up.

**Discussion:**

Findings from this trial will potentially provide valuable evidence to improve physical function and maintain independence among older people from low-resource settings. This will inform health policies and identify locally acceptable strategies to promote healthy aging, prevent and delay functional decline among older Malaysian adults.

**Trial registration:**

ISRCTN22749696.

## Background

The aging population worldwide is fast changing the demographic patterns in societies. Malaysia, as a developing nation, is not exempted from this phenomenon. The proportion of older Malaysians (aged 60 years and above) is projected to double from 6.3% (1.4 million) in 2000 to 12% (4.9 million) by 2030 [1]. The ability to function independently is crucial, as physical disability and functional limitation have profound impacts on public health in terms of healthcare utilisation and long-term healthcare costs and services.

Frailty is increasingly frequent with advancing age, resulting in a spiral of physical and functional decline. While a clear consensus on the operational definition of frailty has yet to be agreed upon [2], the most commonly used definitions of frailty include weight loss, exhaustion, low activity level, slow gait speed and sarcopenia [3]. Frailty involves multiple domains of functioning including mobility, balance, muscle strength, motor processing, cognition, emotions, quality of life, nutrition, endurance and physical activity [4]. Therefore, frail older people are vulnerable to falls, physical disability, loss of independence in performing activities of daily living (ADL), hospitalisation, institutionalisation, increased healthcare costs and mortality [3-5]. Risk factors for frailty in elderly people include comorbidities, physical and psychosocial health, environmental conditions, social factors, nutrition and lifestyle [6].

Assessing measures of frailty among community-dwelling older adults is advocated [4]. Gait speed is a quick, safe, inexpensive and highly reliable tool to predict risk of disability, cognitive impairment, institutionalisation, falls, survival and mortality among older people [7,8]. A walking speed greater than 1.2 m/s predicts high life expectancy [9,10] with 0.8 m/s established as a cut-off point to identify individuals at risk of adverse outcomes [8]. Hairi et al. documented that muscle strength was the single best indicator of age-related muscle changes and is associated with functional limitation and physical disability in ADLs compared to loss of muscle mass and quality [11].

Since functional status is a core determinant of quality of life among older people, interventions that either delay the onset of frailty or attenuate its severity are likely to have cascading benefits for aged adults, their families and society. Multi-faceted approaches comprising an interdisciplinary healthcare team has proven more efficacious in encouraging behavioural change than a single approach in addressing functional difficulties [4], risk of falls [12], number of people who fall and fall rates [13,14] and poor nutrition status [15]; whether home-based [16,17] or in a community setting [18].

Physical activity and exercise are key interventions in improving physical function and maintaining independence in older adults. Physical activity including ADL refers to bodily movement involving muscle contraction and increased energy expenditure whereas exercise refers to planned, structured and repetitive movements aimed at improving various components of physical fitness and encompasses strength, flexibility, balance and endurance [19]. A minimum of 150 minutes per week of aerobic, muscle strengthening and flexibility exercises and physical activity is recommended for older people. If chronic conditions are present, being as physically active as possible is encouraged [20].

The challenge in preventive geriatric care is that physical activity decreases with age and inactive older people are at high risk of metabolic diseases. Many older adults are apprehensive about exercise, which they perceive to be dangerous and may increase their risk of falls. As such, these concerns should be acknowledged and allayed given the safety and health benefits of exercise. Multi-component exercise interventions among frail elderly people have demonstrated significant positive effects in improved physical function [21], balance, mobility and strength in the long-term with continued exercise [18], gait speed [22], reduced risk of falling [12,23], prevented disability in performing ADLs [24] and reduced mobility-related disability [25], maintained independence and reduced hospital admissions [26] and enhanced quality of life [27,28]. Both short and long duration as well as individual and group exercise interventions exhibited a beneficial functional effect [29]. A recommended contact time of at least 2 hours per week for a minimum of 4 weeks with a follow-up period of at least 6 months [26,30,31] have been documented in reviews. High-intensity exercises were more effective than low-intensity exercises with individually tailored physical exercise holding potential long-term benefits [29].

In studies seeking to understand older adults’ motivations and barriers to participate in organised exercise programs, several authors reported that personal motivating factors included group interaction and meeting friends, a break from usual routine and health benefits whereas marketing materials, no out-of-pocket expense and the accessibility of the program were key environmental motivators. Social support factors include socialization and support from and among class participants, family, health care providers and class instructors in addition to exercise content and type of delivery encouraged adherence [32]. Barriers reported were already getting adequate exercise, lack of motivation and discipline, readiness or being unwell or chronically ill, lack of time and facilities and fear of potential for injury and/or falling [33-35]. Gender, education level, age, race, self-efficacy, social support, knowledge of positive attitude toward being physically active were predictors of regular participation in physical activity [34]. That said, more socially-oriented people may be more inclined to join and attend group programmes, thus, a variety of formats need to be readily available in order to maximise engagement in an exercise routine [33].

This study is underpinned by the Health Belief Model which takes a person’s confidence to take action (self-efficacy) into account, their perceived susceptibility to a condition, perceived severity of potential sequelae and evaluation of perceived benefits versus barriers [36]. This model includes the concept of cues to action focusing on the readiness of the individual to take action based on intrinsic or extrinsic factors. Hence, an individual’s values and beliefs markedly impact their engagement in physical activity and adherence rates [37].

Nutrition is an integral part of health and well-being throughout life and is linked to the independence and quality of life of older individuals [38]. Appetite loss, sensory deficit, mastication problems, poor oral health and gastrointestinal disorders often result in inadequate food intake and poor nutrient absorption [39]. Malnutrition among the aged is a major concern and is associated with a progressive decline in health, loss of independence, increased length of hospital stay and readmissions, healthcare costs, fall risks, delayed wound healing, poorer quality of life and mortality [39,40]. The nutritional status of older people is further exacerbated by unintentional weight loss, limited mobility, loneliness, neglect, economic constraints, presence of multi-morbidities and polypharmacy [41]. Nutritional screening among the elderly is useful to identify those at risk of malnutrition who may benefit from appropriate interventions [42]. Nutrition education and counselling interventions involving active participation, goal setting, self-efficacy and peer support has yielded promising results in community-dwelling older adults [43]. The World Health Organization Global Oral Health Programme has also called for oral health promotion among older people to improve their nutritional status and quality of life [44].

Maintaining adequate levels of physical activity and sustaining an appropriate diet are important to minimise the adverse physiological changes associated with aging. Optimal multicomponent exercise and lifestyle interventions to improve physical function and maintain independence among older people in low-resource settings particularly in Asian populations have yet to be established. To address this gap, this study aims to develop and evaluate the effectiveness of a multiComponent Exercise and theRApeutic lifeStyle (CERgAS) intervention program targeted at improving physical performance and maintaining independent living as compared to general health education among older people in an urban poor setting. This study also seeks to identify the subgroup of older people that will benefit the most from this intervention.

## Methods

### Study design and setting

**Figure 1:**
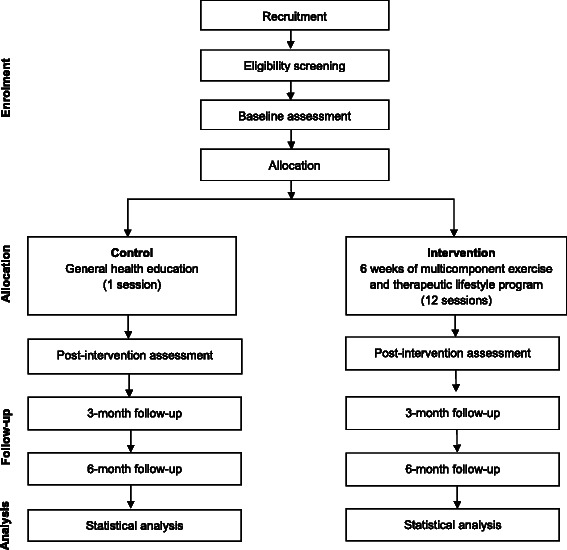
**Overview of CERgAS study flow.** Adapted from the CONSORT 2010 flow diagram.

The CERgAS trial is a 6-week multi-site, single-blind, two-armed, cluster randomised controlled trial with a 6-month follow-up period (Figure 1). The trial comprises a multicomponent exercise and therapeutic lifestyle program for urban poor older people aged 60 years and above. *Cergas* means active in the local Malay language. This study is designed, conducted and reported following the Consolidation Standards of Reporting (CONSORT) 2010 Statement and its extension to cluster randomised trials [45].

This study is conducted among the urban poor living in the Klang Valley, a bustling cosmopolitan area covering 10 municipalities (Kuala Lumpur, Klang, Kajang, Subang Jaya, Petaling Jaya, Selayang, Shah Alam, Ampang Jaya, Putrajaya and Sepang) [46], a symbol of the nation’s progress with rich cultural heritage. Against this backdrop of 7.2 million individuals, lies an easily forgotten group: the urban poor and a growing aging population. In view of this, the Ministry of Urban Wellbeing, Housing and Local Government developed low-cost public subsidised high-rise flats (5 to 18 floors) for the resettlement of squatters and to provide housing to the economically disadvantaged individuals (defined as those with a monthly household income of below RM 2500 or approximately USD 767) [47]. These low-cost public housing projects range from single to 3-bed-room units (650sf), available for lease at a rate of RM 55 (USD 17) to RM 218 (USD 68)/month depending on the size of the units.

The study protocol has been reviewed and approved by the Medical Ethics Committee University Malaya Medical Center (UMMC) (MEC Ref No. 20146–341). Patient information sheets will be explained and provided. Written informed consent will be sought from all study participants prior to the commencement of the study.

### Recruitment

Eligible clusters which refer to low-cost public subsidised flats with a common facility area or hall suitable for exercise sessions and with at least 100 residents aged 60 years and older will be invited to participate in this study. These participants included those who had daily work, whether, shift-work, casual employment or self-employed.

### Eligibility screening

Individuals that agree to participate in the study will be requested for their written informed consent and subsequently be screened to see if they fit the inclusion criteria that is aged 60 years and older, residing in low-cost flats in the Klang Valley and living independently at home. The medical history including medications of each participant was interview-administered and recorded as part of the screening process. Exclusion criteria include older people who are already involved or participating in any structured exercise programme, cognitively impaired and having uncontrolled medical condition(s).

The screening will comprise six measurements and physical function tests to identify eligible older adults for the intervention trial. These measurements will include height, walking speed assessment (walking 4 meters unaided), grip strength (Jamar hand dynamometer), body composition analysis using a 4-point bioelectrical impedance analysis (BIA) equipment (TANITA TBF-300A, Tanita, Japan). Tokens in cash and/or kind including rice packs and household items will be given to participants during screening, baseline, intervention and follow-up measurements to encourage and retain participation.

Eligible participants for the CERgAS intervention trial must be willing and able to attend a one-hour session, twice each week for 6 weeks, have a walking speed slower than 1.24 m/s for females or slower than 1.33 m/s for males [48], not suffering from contraindications to exercise including unstable cardiovascular disease, uncontrolled chronic medical conditions, recent fractures and musculoskeletal diseases that would interfere with the safety and conduct of the intervention program. Once individuals have been assessed as being eligible to participate, they will be invited to attend a baseline data collection.

### Measurements

The following measurements and the physical function tests as aforementioned will be performed at baseline, immediately post-intervention, three and six months at follow-up at a common facility area or hall suitable for exercise sessions at each of the 8 flats.

#### General health status

A face-to-face interview will be conducted to obtain information about participants’ demographics, medical history, falls history, falls efficacy scale (FES-I) [49], cognitive assessment (Mini Mental State Examination (MMSE)) [50], quality of life assessments (SF-12) [51,52], depressive symptoms (Geriatric Depression Scale (GDS) [53]), social support (DUKE Social Support Index [54] and Lubben Social Network Support-6 (LSNS-6) [55]), physical activity (Physical Activity Scale for the Elderly (PASE)) [56], pre-activity readiness questionnaire measured though PAR-Q+ questionnaire [57], stage of motivational readiness for change using the Physical Activity Stages of Change Questionnaire [58], activities of daily living (Katz-ADL) [59] and Lawton’s Instrumental Activities of Daily Living (IADL) [60], nutrition status (Mini Nutritional Assessment (MNA)) [61] and oral health (Geriatric Oral Health Assessment Index (GOHAI)) [62].

#### Anthropometry

Body height will be measured without shoes to the nearest 0.1 cm with a portable stadiometer (SECA 217, Hamburg, Germany). Waist circumference will be measured with a flexible measuring tape (SECA 203, Hamburg, Germany) to the nearest 0.1 cm at the umbilicus, between the tenth rib and the iliac crest. Body composition including fat mass and muscle mass will be assessed through bioelectrical impedance analysis using a portable body composition analyser (TANITA TBF-300A, Tanita, Japan). Resting blood pressure will be also be measured (OMRON HEM-907).

### Randomisation and allocation concealment

Randomisation takes place at the cluster level, that is, the low-cost public subsidised flats, to avoid contamination. The eight housing projects will be randomised into the intervention arm (4 flats) and control arm (4 flats). The study statistician using a computer generated random number sequence will allocate the low-cost flats into the intervention and control group. The statistician using sealed envelopes will advise the study coordinator of the low-cost flats allocation. Allocation will be concealed from the research team recruiting the flats and participants, as randomisation will only take place after baseline measures have been accomplished.

### Intervention

CERgAS is a six-week multicomponent exercise and therapeutic lifestyle training for older people living in low-cost government subsidised flats in the Klang Valley. This intervention programme has been developed in consultation with a trained exercise physiologist and sports science specialist (ASC), a nutritionist (DAL), a dietician (NG) and a dentist (ZA) and will be delivered by an interdisciplinary research team comprising a fitness instructor (ASC), a nutritionist (DAL), a dietician (NG), a nurse and trained counsellors (MFM and MAIAH). The behavioural change strategy of this program is based on the Health Belief Model [63]. A feasibility study involving the older adults and study co-ordinators will be conducted prior to the commencement of the intervention programme to guide the design and implementation of the intervention.

The main mode of delivery is through group exercise sessions with therapeutic lifestyle training on nutrition and oral care education as well as ongoing support by the intervention team. Each session will be an hour long, twice weekly for a duration of six weeks, as recommended in the current body of evidence [26,30,31].

### Multicomponent exercise programme

Participants randomised to the intervention group will attend bi-weekly, 30-minute sessions of supervised exercise, standardised across all clusters in a common facility area for a duration of six weeks. All exercise sessions will be conducted together by a qualified fitness instructor and four trained exercise leaders. The exercise consists of strength, motor fitness and cardiovascular but not endurance. All sessions will last for half an hour and begin with a 5 minute warm-up and stretching sessions for major muscle groups, followed by 20-minute cardio exercise and a 5 minute cool-down period. The warm up and cool down periods will be accompanied by music. Flexibility exercises incorporating both static and dynamic stretching aimed at improving the range of motion, physical performance and reduce risk of injury will include the neck, shoulder and upper arms, chest, back, ankle, hip and lower back. Strength training (hand grip, overhead arm raise, arm curls, wall push-up, back leg raise, side leg raise, knee curl, leg extension and toe stand) will incorporate weight bearing exercises that involves major muscle groups. Balance exercises targeting the lower limbs (stand-on-one-foot, heel-to-toe walk and balance walk) will be performed to enhance stability and improve muscle strength. Sessions will focus on motor fitness involving mobility, dynamic movement, balance and coordination. The aerobic component will be choreographed movements accompanied by music. These repetitive and task-related exercises designed require no special equipment, are easy to learn and are specially designed so that it can be performed without professional supervision at home. The exercise component is designed to include variations and gradual progression to higher intensity. Performing exercises safely will be emphasised during all sessions. Maintaining safety while exercising will be the main consideration at all times. Safety will also be emphasised when the level of difficulty or intensity increases. Participants will be encouraged to walk for 30 minutes at least twice a week to build endurance.

Participants will also receive a CERgAS kit containing home exercise pamphlets illustrating simple, self-guided home-based exercise movements and a DVD to use on the rest of the days. These home exercises are designed to encourage participants to become more physically active by suggesting a variety of ways to incorporate exercise into their daily routine. A booklet on safety precautions, instructions and photographs of home-based exercises will be distributed to all participants. Both the home-based exercise movements and safety instructions will be described using simple language without jargon, printed in 12–14 point text size and ensuring adequate contrast between different colours on the text or pictures and the background.

The researchers will keep a record of the participants’ attendance during all exercise sessions. Intervention group participants will be followed up by telephone calls if they are absent for two consecutive classes. In order to maximise participant contact and follow-ups, participants in both groups are asked to provide at least two sets of contact details.

### Therapeutic lifestyle training

The therapeutic lifestyle training will consist of 30-minute sessions on nutrition education (6 sessions) and oral care (2 sessions). These sessions will complement the exercise programme.

#### Nutrition and oral care education

The nutrition component will consist of modules on healthy eating for older adults, overcoming eating problems (mastication, dry mouth, loss of appetite or taste), eating well on a budget, increasing fruit and vegetable intake, keeping diabetes and hypertension under control (reducing salt and sugar intake) and a heart-healthy diet (reducing fat intake), all designed based on recommended dietary guidelines.

Each session will be conducted by a dietician or a nutritionist and will begin with a 5-minute introduction to the topic and learning objectives. A 10-minute mini-talk designed to convey short and simple key messages will ensue. The talk will be interspersed with hands-on activities, games, quizzes and handouts with an accumulated duration of 10-minutes to reinforce the learning objectives and encourage practical application in daily living. Active participation in activities will be encouraged and rewarded with household items and/or cash. A recap of the lesson and goal-setting will conclude each session. Every session is intentionally designed to build skills that encourage behavioural change including awareness, skills and self-confidence, peer support, goal-setting, overcoming barriers and maintaining lifestyle change. Participants will be provided with lesson summary sheets compiled in a booklet given included their CERgAS kits. These summary sheets will include legible and simple tips to encourage healthy eating.

In the oral care education component, general tips on oral health care for the elderly will be covered.

### On-going support

The aim of the on-going support is to motivate the participants to enjoy movement. The importance of motivation and social support in encouraging participation and adherence in exercise programmes among the elderly has been well-documented [32-35]. To achieve this, the group exercise sessions are designed to be as attractive and pleasurable as possible. This will be conducted over 4 sessions, during the first and final week of the training session. During this time, performance of exercises is not the main focus as yet. The aim will be to encourage and provide as many stimuli as possible to sedentary participants to develop their interest in physical activity. The goals of the second week onwards will shift towards more performance-focused.

### Control group

Participants in the control arm will be provided with a general health education booklet containing information on healthy lifestyle. Participants will be given the similar number of contact hours with different contents not related to physical activity, nutrition or motivation towards healthy living and will be advised to maintain their current level of physical activity.

### Outcomes

#### Primary and secondary outcomes and measurements

The primary outcome of the study is improvement in physical performance among older people assessed by ADL and IADL, grip strength and gait speed. Secondary outcomes will include body composition analysis using BIA along with other measurements consisting of pre-activity readiness for physical activity (PAR-Q+), Physical Activity Scale for the Elderly (PASE), quality of life (SF-12), Falls Efficacy Scale-International (FES-I), fear of falling, depression (GDS), cognitive function (MMSE), nutrition status (MNA) and oral health (GOHAI).

Adverse events will be monitored by the team at the end of each session. Every week participants will fill in a form to indicate if they developed pain, muscle soreness as well as significant injuries or a medical event that results in participant seeking medical attention from a healthcare professional. If participants develop any acute symptoms or any serious adverse events and their condition becomes too serious for the medical doctor in the research team to manage in a community setting, they will be immediately referred and brought to the nearest hospital.

### Blinding

The exercise instructors and team members will be aware of the allocation of participants. However, all outcome measures will be collected by a blinded assessor, unaware of group allocation. Participants will be told not to inform the assessor of their intervention status.

### Sample size

The sample size has been estimated for the primary outcome of this study, that is, changes in physical function. The sample size calculation is estimated based on several measures of physical function including mobility, balance, stability (8-Foot Up and Go Test), aerobic endurance (6-minute walk test) and activities of daily living (ADL). In order to achieve 80% power to detect differences in the 8-Foot Up and Go Test, 6-minute walk test and ADL as previously reported [27,64,65], an individually randomised trial would require 45 participants per arm. With a hypothesised intra-cluster correlation coefficient (ICC) of 0.02 and a minimum of 20 participants recruited per cluster, the required sample size is 63 per arm. Assuming 30% loss to follow-up, 82 participants per arm or a minimum total of 164 participants will be required. To recruit at least 164 participants with a cluster size of approximately 21, it will be necessary to recruit 8 low-cost flats.

### Process evaluation

A range of process data will be conducted to complement the outcome data. Process measures will include participants’ attendance at exercise and training sessions, participants’ satisfaction with all three intervention components (group exercise session, therapeutic lifestyle training focusing on nutrition and oral care and on-going tailored motivation support). Participants’ general satisfaction with the program, intensity, duration and pace of the program will be evaluated.

A formal effectiveness evaluation will be conducted via focus-group discussions with key stakeholders, including cluster community leaders, study co-ordinators, project officers and study participants after the intervention to assess the implementation of the intervention, to evaluate the sustainability and identify enablers and barriers. In addition, a cost-effectiveness of the intervention will be evaluated based on the total cost of the intervention and the number of participants.

### Statistical analysis

The study design provides for four assessments with study participants – at baseline, immediately post-intervention, three months and six months follow-up. Description of participants’ baseline characteristics will be reported by groups. Categorical variables will be summarised by frequencies and percentages. Continuous variables will be summarised by means and SD or non-parametric equivalents. The data analysis for this trial will be undertaken on an intention-to-treat (ITT) principle [66]. The continuous variable outcomes (immediately post-intervention, three months and six months) will be compared between groups using a generalised estimating equations and linear mixed effect models with adjustment for baseline covariates. Groups with dichotomous outcomes will be compared using logistic regression models. Baseline values will be entered as covariates. Statistical significance is set at p < 0.05 and mean 95% confidence intervals (CI) or differences in percentage between the two groups will be reported at the follow-up time points. Data will be entered and analysed with SPSS 22.0 (SPSS Inc., Chicago, IL, USA).

### Study status

Data collection commenced in October 2014 and is on-going. Screening and baseline measurements have been completed and the intervention trial is currently being conducted.

## Discussion

There is an urgent need for locally acceptable sound interventions to promote healthy aging among older Malaysians. The CERgAS trial presents a unique approach, delivering a multicomponent intervention to older adults aged 60 years and older in an Asian urban poor setting. Furthermore, this study is an important step forward and contributes to establishing the effectiveness of a multicomponent exercise and therapeutic lifestyle intervention to increase physical activity and improve nutrition status among community-dwelling older adults. In addition, the demographics of participants who attend the study will be identified and the extent of acceptance towards the intervention according to demographic characteristics such as gender, age and ethnicity will be examined. Moreover, this community-based lifestyle intervention will be conducted in a naturalistic setting, allowing participants to integrate changes into their daily lives in a real-life context. This implies that the intervention delivered can be potentially transferable to other community settings. The process evaluation will provide insight as to how the findings can be extrapolated to urban and rural settings.

While every effort will be made to maximise participation rates in each cluster, this study is limited by the small number of clusters involved in the study and may present as confounding issues during the analyses.

Improved gait speed, muscle strength, quality of life, ADLs and reduced falls accrued from regular participation in exercise and physical activity are anticipated. Adopting a healthy and nutritious diet along with good oral health will likewise improve nutritional status, functional ability and ultimately enhance their quality of life. Finally, the on-going motivational support will provide the elderly adults with the support and encouragement needed to reach their goals, identify barriers and suggest ways to overcome obstacles in efforts to increase their self-efficacy towards healthier aging. To encourage adherence in the intervention, each component (exercise, nutrition and oral care) will be designed to be interactive, enjoyable and manageable.

If the intervention produces significant positive effects, the findings will provide valuable evidence and serve as a model to inform health policies and identify locally acceptable strategies to promote healthy aging and prevent and delay functional decline among older Malaysians.

## Abbreviations

ADL: Activities of daily living
FES-I: Falls Efficacy Scale-International
MMSE: Mini Mental State Examination
SF-12: Short Form 12
GDS: Geriatric Depression Scale
LSNS-6: Lubben Social Network Support-6
PASE: Physical Activity Scale for the Elderly
PAR-Q+: Physical Activity Readiness Questionnaire for Everyone
IADL: Instrumental activities of daily living
MNA: Mini Nutritional Assessment
GOHAI: Geriatric Oral Health Assessment Index
